# Ecological interactions between Gulf of Mexico snappers (Teleostei: Lutjanidae) and invasive red lionfish (*Pterois volitans*)

**DOI:** 10.1371/journal.pone.0206749

**Published:** 2018-11-01

**Authors:** Anthony R. Marshak, Kenneth L. Heck, Zachary R. Jud

**Affiliations:** 1 Department of Marine Sciences, University of South Alabama, AL, United States of America; 2 Dauphin Island Sea Lab, Dauphin Island, AL, United States of America; 3 Marine Sciences Program, Department of Biological Sciences, Florida International University, North Miami, FL, United States of America; Department of Agriculture and Water Resources, AUSTRALIA

## Abstract

Indo-Pacific red lionfish (*Pterois volitans*) have invaded the western Atlantic, and most recently the northern Gulf of Mexico (nGOM), at a rapid pace. Given their generalist habitat affinities and diet, and strong ecological overlap with members of the commercially valuable snapper-grouper complex, increased density and abundance of lionfish could result in significant competitive interactions with nGOM commercially important species. We experimentally investigated the intensity of behavioral interactions between lionfish and indigenous, abundant and economically important juvenile nGOM red snapper (*Lutjanus campechanus*), and other increasingly abundant juvenile tropical snapper species (gray snapper—*L*. *griseus* and lane snapper—*L*. *synagris*) in large outdoor mesocosms to examine snapper vulnerabilities to lionfish competition. When paired with lionfish, red snapper swimming activity (i.e., time swimming and roving around experimental tank or at structure habitat during experiments) was significantly lower than in intraspecific control trials, but gray and lane snapper swimming activities in the presence of lionfish did not significantly differ from their intraspecific controls. Additionally in paired trials, red and lane snapper swimming activities were significantly lower than those of lionfish, while no significant difference in swimming activities was observed between lionfish and gray snapper. We found that red snapper prey consumption rates in the presence of lionfish were significantly lower than in their intraspecific 3-individual control trials, but when paired together no significant differences in prey consumption rates between red snapper and lionfish were observed. When paired with lane or gray snapper, lionfish were observed having comparatively higher prey consumption than snappers, or as observed in lionfish intraspecific 1-individual controls. However, lane and gray snapper consumption rates in the presence of lionfish did not significantly differ from those in intraspecific controls. These findings suggest that competition between juvenile snappers and invasive lionfish may be variable, with lionfish exhibiting differing degrees of competitive dominance and snappers exhibiting partial competitive vulnerability and resistance to lionfish. While the degree of intensity at which these interactions may occur in nGOM reefs may differ from those observed in our findings, this study enables greater understanding of the potential ecological effects of red lionfish on native reef fishes.

## Introduction

Increasing anthropogenic impacts in marine ecosystems have raised concerns about the future of their ecological stability, as they continue to be affected by a suite of stressors that includes climate change, overfishing, and marine pollution [1–3]. Compounding these factors is the introduction of non-native invasive species [4–5]. While their long-term impacts on marine biodiversity remain uncertain, non-native species clearly have the potential to alter the ecology of invaded habitats [6–7]. For example, studies frequently suggest that non-native species are either better competitors or predators than native species [8–9], and invasions have led to habitat displacements of resident fauna [10], localized reductions in prey, and shifts in species composition [11–12]. In some cases, however, the effects of introduced species may be less pronounced, owing to competitive superiority of natives [13], and diluted effects of invaders in more speciose environments [14]. These mixed observations reflect the ambiguity that remains in understanding the biological effects of non-native species on residents. Ultimately, however, pronounced ecological effects of predatory invaders are anticipated [15–16], resulting in both direct and indirect effects on food webs.

Owing to their high fecundities, strong dispersal abilities, early maturity, and generalist diets [17], Indo-Pacific red lionfish (*Pterois volitans*) have invaded the western Atlantic, Caribbean Sea, and more recently the Gulf of Mexico [18]. Average lionfish densities in invaded regions are estimated to reach 102 per hectare (ha), which are much greater than those in their native ranges (average: 25 per ha) [19]. Because of their life history traits, and oceanographic patterns favoring dispersal [20], lionfish rapidly expanded into the northern Gulf of Mexico (nGOM) in 2013 [21], where individuals colonized natural hardbottoms (50–200 m depth), grew to large sizes [22], established very high densities on artificial reefs [23], and subsequently expanded into shallower habitats (2–40 m depth) [24]. Given their limited home ranges [25] and generalist trophic nature [26], lionfish have depleted demersal prey fishes and benthic invertebrates over short time frames [27–28], exhibiting faster growth and higher consumption rates than other mesopredators [29]. With their rapid proliferation, red lionfish are anticipated to have negative effects on nGOM fish assemblages, including small cryptic reef fishes and the commercially valuable snapper-grouper complex [23, 30].

While there have been many studies of the predatory effects of lionfish in its invaded range [27, 29, 31], investigations into their competitive interactions with native species have occurred only recently. Some studies have suggested high potential for lionfish to exploitatively outcompete other mesopredatory reef fishes [18, 32]. In the Caribbean Sea, for example, lionfish caused three times the mortality to native prey fishes than the ecologically similar coney (*Cephalopholis fulva*) [29], and unpublished referenced data [18] estimated lionfish consumption at two times the rate of similar graysby (*C*. *cruentata*). However, based upon emerging investigations, strong competitive interactions between lionfish and native fish species at larger scales do not appear to occur [33], and limited measurable effects on prey fish communities have been observed [34]. While evidence of lionfish population control by larger predatory fishes remains uncertain [35], the presence of red grouper (*Epinephelus morio*) has been suggested to ameliorate lionfish predation [36]. Additionally, partial habitat displacement of lionfish by grouper [37] and spiny lobster [38] has been observed, in addition to apparent partial niche differentiation with smaller groupers [39]. A large-scale relationship between lionfish and grouper has not been detected [33]. However, based on stable isotope data, lionfish do appear capable of directly competing with Nassau grouper (*Epinephelus striatus*) for food resources, given their similar spatial and trophic niche overlaps during their life histories [40]. Lionfish do not exhibit flight responses common to other fish species, but instead adopt more bold postures and behaviors [29], suggesting potential competitive advantage, although their impact beyond small patch reef habitats and on native fauna in their invaded range remains unclear [41].

Although the potential for red lionfish to outcompete native reef fishes in other regions still remains uncertain, based upon lionfish predatory abilities and their habitat and dietary overlap with mesopredatory snappers [23], we hypothesized that lionfish would be more active and consume more prey than native nGOM snapper species. The objectives of this study were to document behavioral interactions between lionfish and co-existing juvenile nGOM snapper species (gray snapper–*Lutjanus griseus*, lane snapper–*L*. *synagris*, red snapper–*L*. *campechanus*) to determine their comparative vulnerabilities to red lionfish competition. These snappers co-occur in nGOM offshore hardbottom and structured reef habitats where space and prey resources are both limited [42], and have been observed interacting during similar times of day for shared resources [42–47]. Given their habitat and prey overlap with red lionfish [23, 48], and presumed effects of lionfish on their densities [49], competition between snappers and lionfish for shared resources is expected to be high.

## Materials and methods

### Ethics statement

This study was conducted in accordance with the laws of the States of Alabama and Florida and under IACUC protocols (Permit # 05043-FSH) approved by the University of South Alabama and the Dauphin Island Sea Lab. A Special Activity License (SAL-11-1334-SR) issued by the state of Florida was additionally used for collecting and transporting juvenile lane and gray snapper. All efforts were made to minimize stress and to ensure care while capturing and transporting animals, and when using them in experiments.

### Experimental methods

To examine competitive interactions between lionfish and nGOM snapper species, we conducted a series of experimental trials in mesocosms. Control and interaction experiments were used to quantify inter- and intraspecific competitive intensities among snapper species and lionfish at the Dauphin Island Sea Lab outdoor mesocosm facility from 29 June to 25 October 2012 and 22 April to 11 August 2013. Juvenile and early adult red (170–350 mm total length; TL) and lane (105–350 mm TL) snapper were collected in offshore natural and artificial reef habitats up to 40 miles south of coastal Alabama using both buoyed small chevron traps (maximum dimensions: 0.7 m x 0.6 m x 0.29 m; 0.635 cm mesh) and hook-and-line. Juvenile and early adult gray snapper (150–313 mm TL) were collected using fish traps (maximum dimensions: 1.2 m x 1.2 m x 0.61 m; 2.54 cm mesh) and hook-and-line in natural reef and seagrass habitats south of Layton, Florida, and via hook-and-line near Mobile Bay Industrial Canal in Theodore, Alabama. Lionfish (135–228 mm TL) were collected by netting individuals while snorkeling or scuba diving in south Florida. All fishes were transported live in aerated or oxygenated coolers from their collection sites to the outdoor Dauphin Island Sea Lab mesocosm facility or indoor wetlab, where they were allowed to acclimate in holding tanks for two weeks prior to initiation of experiments.

During acclimation and holding periods, all snappers and lionfish were fed 0.25 kg (per tank) of squid (*Loligo* sp.) twice weekly, following previous mesocosm investigations using red snapper [42, 50]. Lionfish were additionally fed twenty juvenile blue crabs (1.0–2.5 cm carapace width) every other day, given lionfish predatory feeding demands [51–52]. All individuals used in trials were starved for 24 hours prior to the initiation of experiments. Previous studies [48, 53] have identified squid and crustaceans as significant components of the diets of juvenile and post-juvenile snappers, while additional investigation [23] has identified squid and crustaceans, including crabs, as a significant component of nGOM lionfish diets.

Gray, lane, and red snapper behavioral and consumptive interactions with lionfish were documented in mesocosms (2.4 m diameter, 0.9 m height) filled with saltwater to 0.3 m depth, lined with fine sediment and shell substrate, and containing two haphazardly placed concrete blocks (dimensions: 0.39 m x 0.19 m x 0.19 m) as simulated reef habitat. Additive and substitutive designs with interspecies treatments and monoculture controls to account for both species identity and density were employed to address both inter- and intraspecific competitive interactions, and all experimental factors were randomized. Each three-hour experimental trial (Table 1, n = 32) was video recorded with suspended cameras. Trials were performed between the hours of 1000 and 1930. While snappers and lionfish have been documented undertaking crepuscular activities [31, 54–56], these species have likewise been observed interacting and feeding in their natural habitats during the day [44–47, 49, 57]. Previous investigation [42] has additionally shown interactions among the three snapper species as being pronounced during these times of study, while lionfish have been classified as diurnal feeders [51].

**Table 1:** Experimental mesocosm combinations assessing interactions between juvenile red snapper, lane snapper, gray snapper, and lionfish.

	Density of fishes			
Treatment	1 per tank	3 per tank	4 per tank	Total replicates
Gray snapper control		n = 3	n = 2	n = 5
Lane snapper control		n = 3	n = 3	n = 6
Red snapper control		n = 4	n = 3	n = 7
Lionfish control	n = 3		n = 3	n = 6
Gray snapper-Lionfish			n = 3	n = 3
Lane snapper-Lionfish			n = 3	n = 3
Red snapper-Lionfish			n = 2	n = 2
**Total = 32 replicates**				

Combinations are listed according to trials (n = 3) between lionfish and the three snapper species. In total, 8 trials were run for the paired species combinations (3 snapper and 1 lionfish), in addition to 24 monoculture control trials to account for snapper and lionfish species identity (n = 3 individuals for snapper, n = 1 individual for lionfish) and density (n = 4 individuals for snapper, n = 4 individuals for lionfish) of the total number of individuals observed per treatment.

To approximate natural offshore hardbottom fish densities in the nGOM (0.49/m^2^) [23], three representative fish per snapper species and one lionfish were used in each investigation examining interspecific interactions (i.e., 3 gray snapper– 1 lionfish, 3 lane snapper– 1 lionfish, 3 red snapper– 1 lionfish). Corresponding uniformly stocked monospecific control trials for lionfish and each snapper species were undertaken to account for both species identity (n = 3 individuals of a given snapper species or n = 1 individual lionfish, per trial) and density (n = 4 individuals of a given snapper species or n = 4 individual lionfish, per trial).

To examine competitive interactions for shared prey resources in control and snapper-lionfish interaction trials, we examined standardized prey consumption and predatory behaviors. With increasing numbers of offshore lionfish, we theorized that local prey resource limitation could occur at a scale similar to that of our experimental area as predator species richness and density increased on, and adjacent to, limited natural reef habitat. Therefore, ten juvenile blue crabs (*Callinectes sapidus*; 1.0 to 2.5 cm carapace width) were collected offshore by trawling or within nearshore seagrass and marsh habitats by seining, and were used as prey in each experimental trial, independent of snapper or lionfish density. A density of 0.4 crabs/m^2^ (based on 10 crabs and mesocosm area) is much less than published densities of decapod crustacean prey adjacent to or within natural nGOM reef habitats, and simulates prey limitation [58]. Although this design does not factor for prey depletion, additional investigation [59] has highlighted the value of holding initial prey constant to inform about stochastic predation, which may be more likely to occur with increasing predator richness and density. A similar study [42] has demonstrated that juvenile snappers prey upon blue crabs of this size range.

Snappers and lionfish were introduced to treatment tanks in a randomly determined location of entry and sequence per treatment. Some snappers, lionfish, and surviving crabs (~6%) were re-used in subsequent trials; however, this was done non-consecutively and two to five days after that individual’s previous use in trials to allow for re-acclimation. Reuse of animals in experiments occurs occasionally and has been promoted in certain circumstances when allowing for suitable recovery times between behavioral trials [60–61]. Additionally, to minimize bias, individuals were never used together more than once. Snappers and lionfish were allowed to acclimate for one hour in experimental tanks prior to trials, and blue crabs were haphazardly introduced to tanks at the onset of the experiment. As most fishes remained motionless while acclimating, during acclimation periods no interactions or patterns were observed that suggested any direct influence or bias upon subsequent behaviors during experimental periods. Given the large size of the experimental mesocosms, and haphazard placement of prey, snapper and lionfish awareness of initial blue crab location was therefore minimized due to lowered probability of immediate encounter. Prior to each trial, water temperature, salinity, dissolved oxygen, and time of day were recorded. At the conclusion of each three-hour trial, the number of crabs remaining was recorded and all fishes were measured (total length) to the nearest millimeter and weighed to the nearest gram.

The video from each 3-hour trial was analyzed in its entirety to quantify all response variables as defined in S1 Table, which included swimming times at structured habitat and around the mesocosm, lionfish pectoral fin flares, snapper and lionfish interactions (i.e., time swimming together, interspecific approaches toward, and retreats from, fishes), consumption rates, time pursuing prey, predatory attacks, non-consumptive prey approaches, retreats from prey, number of times that fishes ignored nearby prey, and aggressive interactions for lionfish and snapper species during each interaction or control trial. Increased swimming activity by fishes in the presence of other species or conspecifics has been strongly associated with social dominance and aggression in behavioral interactions [62–63], and with successful prey consumption [64]. As quantified behavioral and consumptive variables (S1 Table) did not conform to parametric assumptions (i.e., homoscedacity examined using Levene’s F-test; normality examined using Shapiro Wilk test), all behavioral and consumptive variables were analyzed using one-way Kruskal-Wallis tests (n = 14) and *post-hoc* Conover-Iman multiple comparisons to examine cross-comparative behavioral responses among trial types. Relationships between behavioral variables, environmental parameters, time of day, and fish size were assessed using multiple linear and non-linear regressions [65].

## Results

Average environmental variables (temperature, salinity, and dissolved oxygen) for all trials were 24.3°C ± 0.83 (standard error; SE), 22.1 ± 0.57 (SE), and 7.74 mg/L ± 0.45 (SE), respectively. Specific values for control and interaction trials are listed in Table 2. While lane snapper control trials were performed at cooler (Δ 8°C) temperatures than lane-lionfish interaction trials, no significant differences in temperature were found among any control or interaction trial types (K = 9.75, p = 0.136). Additionally, no significant differences in salinity (K = 11.65, p = 0.07) or dissolved oxygen (K = 6.65, p = 0.354) among trial types were found. Among control and snapper-lionfish interaction trials, average total lengths (mm; Table 3) were similar for gray (U = 86.5, p = 0.606), lane (U = 62.5, p = 0.151), and red snapper (U = 56, p = 0.742). Similar sized lionfish were used in control and red (U = 11.5, p = 0.647) and lane snapper-lionfish interaction trials (U = 29.5, p = 0.449), although by chance significantly larger lionfish (~ 10% larger on average; U = 42.5, p = 0.012) were used in gray-lionfish interaction trials.

**Table 2:** Environmental parameters (±1 SE) in experimental mesocosms during snapper and lionfish control and interaction trials.

Treatment	Temperature	Salinity	Dissolved Oxygen
Gray snapper control	24.17 ± 1.38	22.81 ± 1.04	7.25 ± 0.62
Lane snapper control	20.49 ± 2.62	18.54 ± 1.17	10.92 ± 1.87
Red snapper control	23.68 ± 1.91	22.51 ± 1.02	6.96 ± 0.30
Lionfish control	26.81 ± 0.98	23.64 ± 1.33	7.31 ± 0.22
Gray snapper-Lionfish	21.70 ± 0.70	24.07 ± 0.32	6.11 ± 0.78
Lane snapper-Lionfish	28.83 ± 0.91	20.22 ± 2.32	7.29 ± 0.49
Red snapper-Lionfish	27.35 ± 3.15	24.46 ± 1.46	6.57 ± 1.02

Temperature (^o^C); Dissolved Oxygen (mg/L)

**Table 3:** Total length, weight, and weight/length ratios (±1 SE) of juvenile and early adult gray, lane, and red snappers and lionfish used in control and interaction trials.

Treatment	Species	Total Length	Weight
Gray Control		232.88 ± 15.95	215.29 ± 43.6
Lane Control		183.33 ± 20.48	182.63 ± 84.49
Red Control		253.42 ± 9.04	219.11 ± 30.25
Lionfish Control		171.8 ± 7.50	56.99 ± 8.12
Gray-Lion	Gray	216.22 ± 14.95	156.9 ± 24.02
	Lion	214.33 ± 8.09	121.47 ± 18.86
Lane-Lion	Lane	197.00 ± 22.91	246.67 ± 103.98
	Lion	193.33 ± 3.33	70.00 ± 10.00
Red-Lion	Red	252.50 ± 25.29	200.00 ± 145.26
	Lion	192.50 ± 7.50	N/A

Total length (mm); weight (g)

Due to equipment malfunction, weight data were not taken for lionfish in red

snapper-lionfish interaction trials.

**Fig 1:**
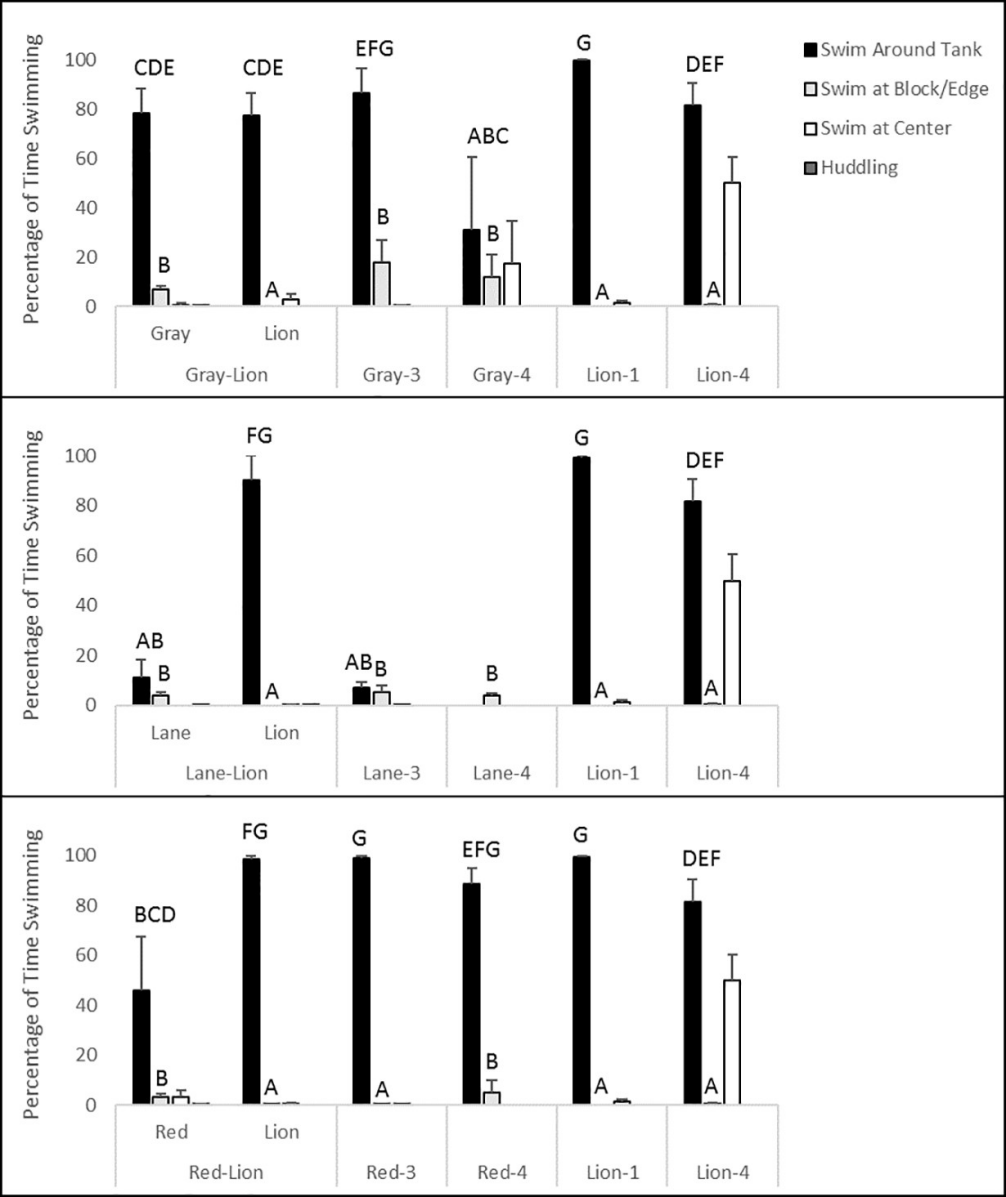
Percentage of time spent swimming around the entire experimental mesocosm (±1 SE), swimming at concrete blocks or edge of mesocosm, swimming at the center of the mesocosm, or huddling at blocks by lionfish and red, lane, and gray snappers during control and interaction experiments. Data in the top panel are from control trials of three and four individuals for gray snapper (gray) and one or four individuals for lionfish (lion), and interaction trials between lionfish (lion) and gray snapper species (gray). Data in the middle panel are from control trials of three and four individuals for lane snapper (lane) and one or four individuals for lionfish (lion), and interaction trials between lionfish (lion) and lane snapper species (lane). Data in the bottom panel are from control trials of three and four individuals for red snapper (red) and one or four individuals for lionfish (lion), and interaction trials between lionfish (lion) and red snapper species (red) Capitalized letters above bars denote significant groupings (p<0.05) in *post-hoc* pairwise comparisons. Behaviors are not mutually exclusive, as individuals of a given species could be observed performing separate swimming behaviors during the same time.

In most cases, swimming times (Fig 1) around the entire mesocosm (K = 32.091, df = 13, p = 0.002) and near blocks (K = 30.039, df = 13, p = 0.005) differed significantly by species within and between control and interaction trials. Most fishes swam predominantly around the entire tank. In interaction trials, lionfish swam significantly more than lane snapper, which demonstrated very little swimming activity in experimental and control trials, and red snapper, which swam at nearly half the activity levels of lionfish in experimental trials. Additionally, no significant difference in swimming times was observed between paired gray snapper and lionfish. Compared to their respective control trials, when lane or gray snapper were paired with lionfish, there were no significant differences in swimming times. However, when paired with lionfish, red snapper swam significantly less often with diminished movements than in their control trials. During interaction trials, lionfish swam significantly less than in control trials when paired with gray snapper. However, this difference only occurred in lionfish control trials with one individual.

Among conspecific control trials, swimming times between trials using three and four individuals were not significantly different for red and lane snapper (Fig 1). However, gray snapper and lionfish were significantly less active in control trials with four individuals than in trials with three individuals (gray snapper) or one individual (lionfish), respectively, indicating intraspecific effects upon activity. Lionfish and gray snapper in 4-fish control trials also spent a higher proportion of time swimming in the center of the tank, although this trend was not significant (K = 19.241, df = 13, p = 0.116; Fig 1).

Lionfish spent little to no time at concrete blocks during control and experimental trials, as they actively swam around the entire tank (Fig 1). However, they did spend the most time at the center during their interactions with gray snapper, although this trend was not significant. Likewise, a similar pattern of fish spending little time at concrete blocks and swimming around the entire tank was observed during control trials containing three red snapper. Although low in proportion, red snapper swimming activity at blocks was still significantly higher during lionfish interaction trials than in controls with three red snapper, but did not differ from four-fish red snapper controls. No significant differences in swimming times at blocks were observed for gray or lane snapper within or between control and interaction trials, although their activities were significantly higher than those of lionfish and three-fish red snapper control trials. Although significantly highest for red snapper in red-lionfish interaction trials, and for gray snapper in control trials of four individuals (K = 18.627, df = 13, p = 0.009; Fig 1), little to no huddling of fishes at blocks was observed.

**Fig 2:**
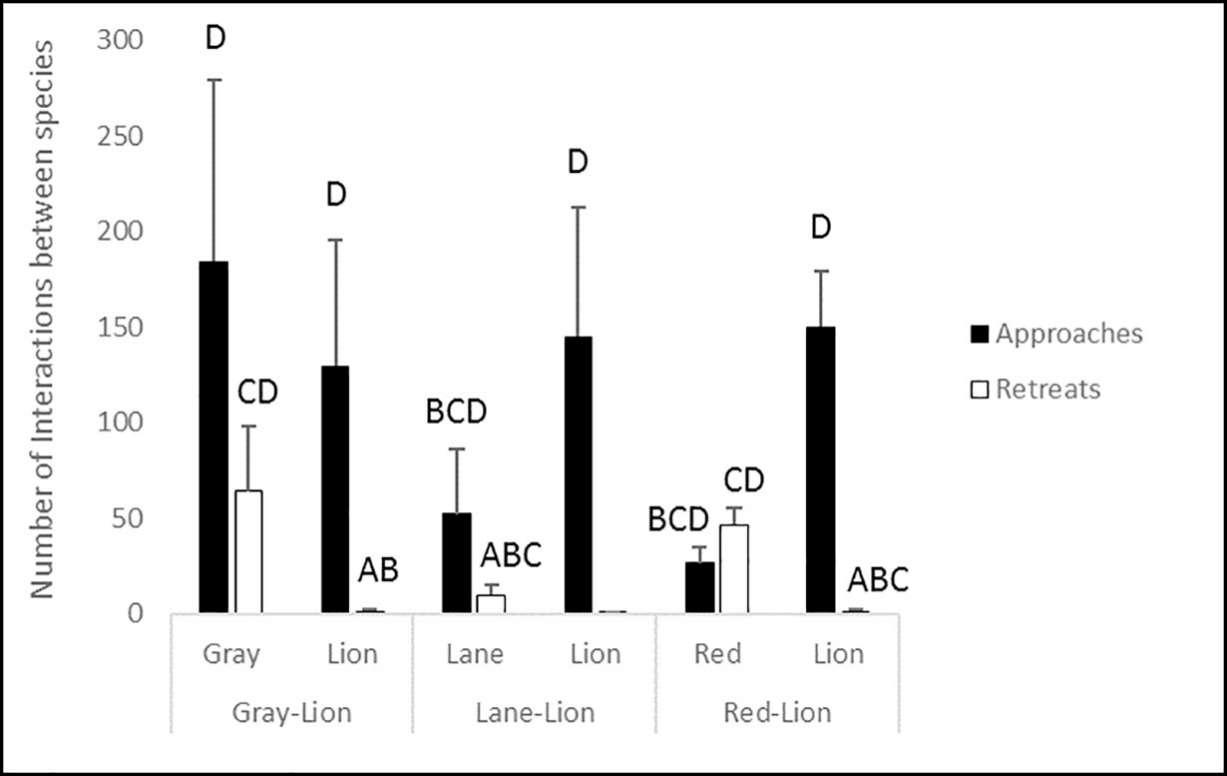
Number of interspecific approaches and retreats on other fish species (± 1 SE) between lionfish and red, lane, and gray snappers during interaction experiments. Capitalized letters above bars denote significant groupings (p<0.05) in *post-hoc* pairwise comparisons.

No significant difference in snapper-lionfish interaction times among treatments was observed (F_2,7_ = 0.509, p = 0.629; Table 4), with the highest observed average percentage of interaction time occurring between gray snapper and lionfish. Additionally, lionfish flared their pectoral fins in the presence of red snapper significantly more often (F_2,7_ = 9.637, p = 0.019) than in the presence of lane or gray snapper. Gray snapper were observed swimming underneath the pectoral fins of lionfish in all three species interaction trials for 98.7 seconds ± 36.2, and were the only species for which this behavior was observed. While no significant differences in the number of interspecific approaches or retreats (Fig 2) between lionfish and snappers were observed, a significantly higher number of approaches than retreats was observed for lionfish in all three experimental snapper-lionfish treatments (K = 20.960, df = 11, p = 0.034). Upon approach by lionfish, gray snapper and red snapper flared fins, and red snapper would often pause swimming.

**Table 4:** Percentage of trial time spent interacting (±1 SE) between lionfish and red, lane, and gray snapper species, and number of lionfish pectoral fin flares, during mesocosm experiments.

Trial	Percentage of time in which species interact	Number of lionfish pectoral fin flares
Gray-Lion	13.06 ± 5.27	3 ± 1.53
Lane-Lion	6.82 ± 5.37	4.67 ± 2.4
Red-Lion	7.17 ± 1.49	20.5 ± 5.5*

*p<0.05

**Fig 3:**
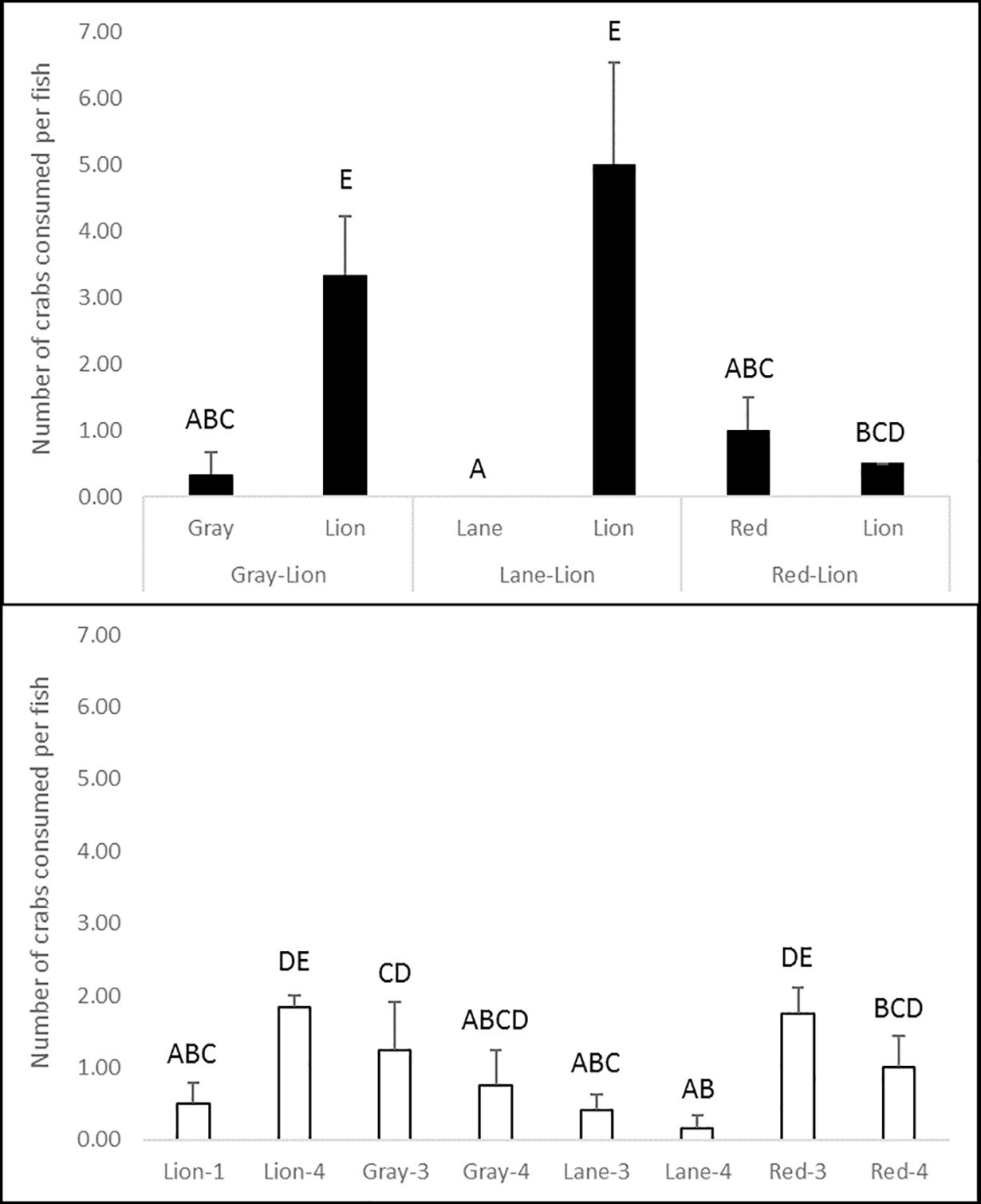
Consumption rates of blue crabs per fish (± 1 SE) by lionfish and juvenile red, lane, and gray snappers during control and interaction experiments. Data in the top panel are from interaction trials between lionfish (lion) and snapper species (gray, lane, or red snapper), and data in the bottom panel are from lionfish and snapper monoculture control trials of three and four individuals of a given snapper species and one or four individuals for lionfish (lion). Capitalized letters above bars denote significant groupings (p<0.05) in *post-hoc* pairwise comparisons. No standard error was observed for lionfish consumption in red snapper-lionfish interaction trials.

**Fig 4:**
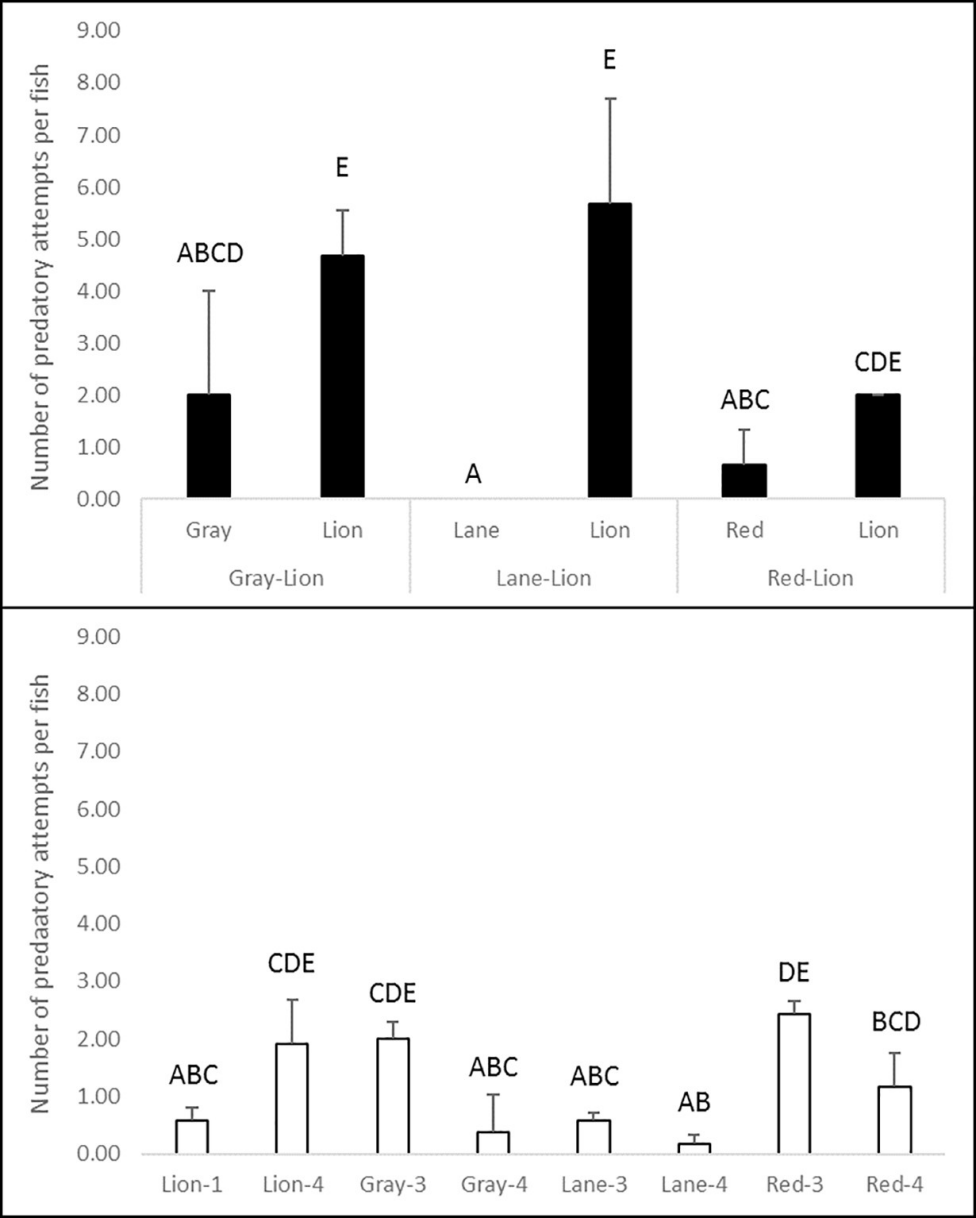
Predatory attempts per fish on blue crab prey (± 1 SE) by lionfish and juvenile red, lane, and gray snappers during control and interaction experiments. Data in the top panel are from interaction trials between lionfish (lion) and snapper species (gray, lane, or red snapper), and data in the bottom panel are from lionfish and snapper monoculture control trials of three and four individuals of a given snapper species and one or four individuals for lionfish (lion). Capitalized letters above bars denote significant groupings (p<0.05) in *post-hoc* pairwise comparisons. No standard error was observed for lane snapper in lane snapper-lionfish interaction trials or for lionfish in red snapper-lionfish interaction trials.

In interaction trials, prey consumption (K = 23.688, df = 13, p = 0.007; Fig 3) and predatory attempts (K = 26.099, df = 13, p = 0.016; Fig 4) per fish were significantly higher for lionfish than for lane or gray snappers, although not for red snapper. No significant differences in consumption rates or predatory attempts were observed between lane or gray snappers in control and interaction trials. Red snapper in lionfish interaction trials attempted to prey upon and consumed significantly fewer crabs than those in three-individual trials, but not four-individual trials. Lionfish in trials with lane and gray snapper attacked prey more readily and consumed significantly more crabs than in one-individual, but not four-individual control trials. However, when paired with red snapper, no significant difference in lionfish consumption was observed as compared to control trials. Additionally, no significant difference in lionfish predatory attempts was observed between interaction and control trials. Lionfish consumed significantly fewer crabs when paired with red snapper than when paired with tropically-associated snappers, and significantly higher numbers of crabs per fish in four-individual than in one-individual control trials. However, there were no significant differences in predatory attempts among these combinations. Occasionally, red snapper were observed attacking and consuming crab prey that was initially chased by lionfish toward structure where they rested. However, these behaviors were uncommon.

**Fig 5:**
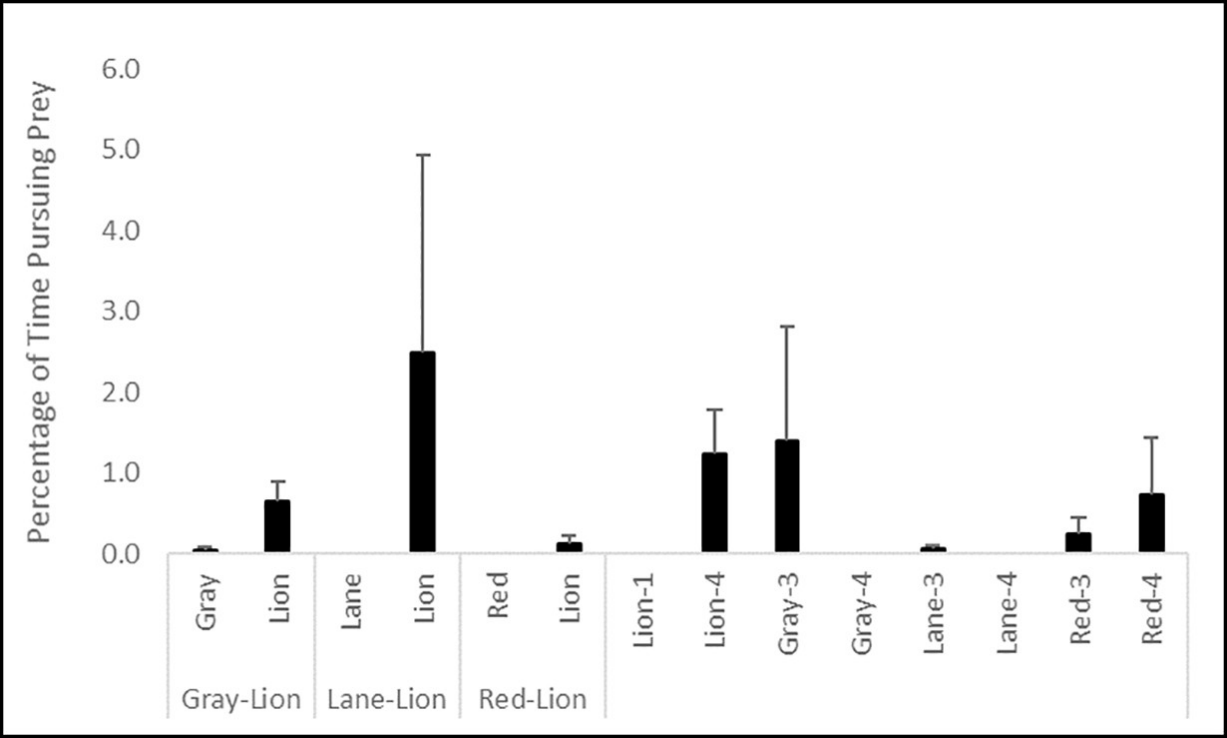
Percentage of time pursuing blue crab prey (±1 SE) by lionfish and red, lane, and gray snappers during control and interaction experiments. Data in the left portion of the figure are from interaction trials between lionfish and a given snapper species, and data in the right portion of the figure are from lionfish and snapper monoculture control trials of three and four individuals of a given snapper species (gray, lane, or red snapper), and one or four individuals for lionfish (lion).

**Fig 6:**
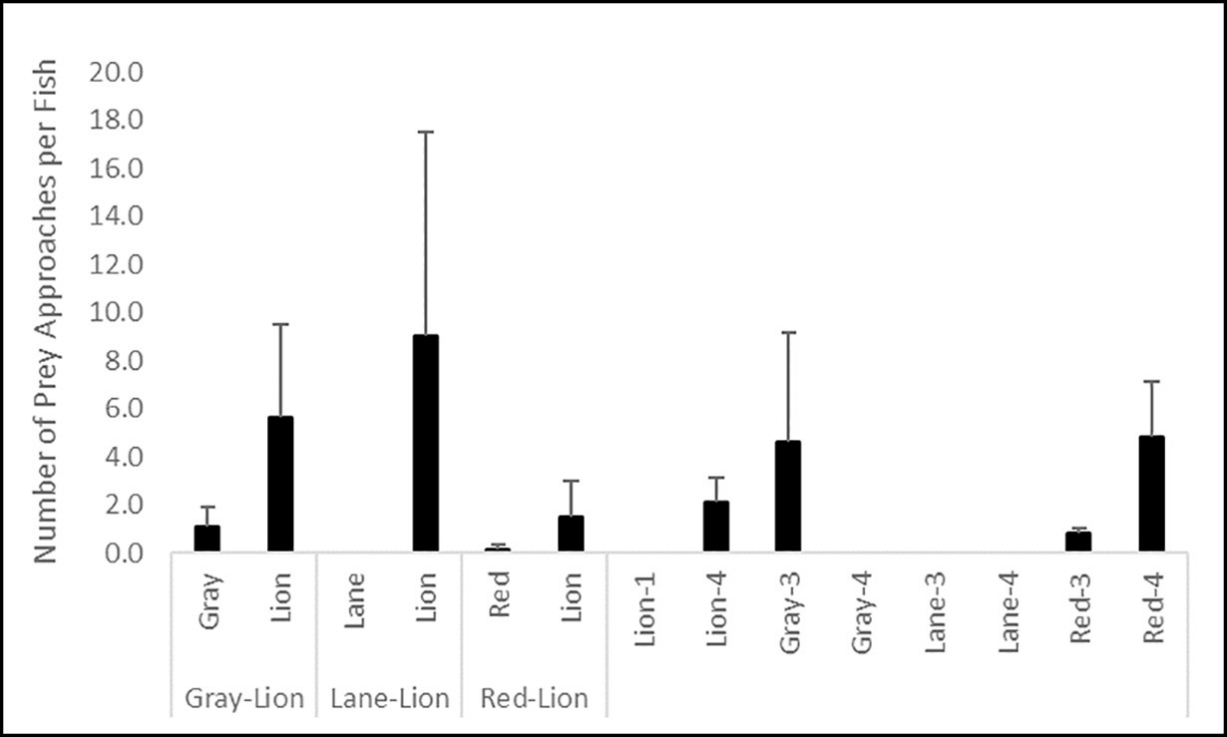
Number of non-consumptive approaches per fish on blue crab prey (± 1 SE) by lionfish and red, lane, and gray snappers during control and interaction experiments. Data in the left portion of the figure are from interaction trials between lionfish and a given snapper species, and data in the right portion of the figure are from lionfish and snapper monoculture control trials of three and four individuals of a given snapper species (gray, lane, or red snapper), and one or four individuals for lionfish (lion).

**Fig 7:**
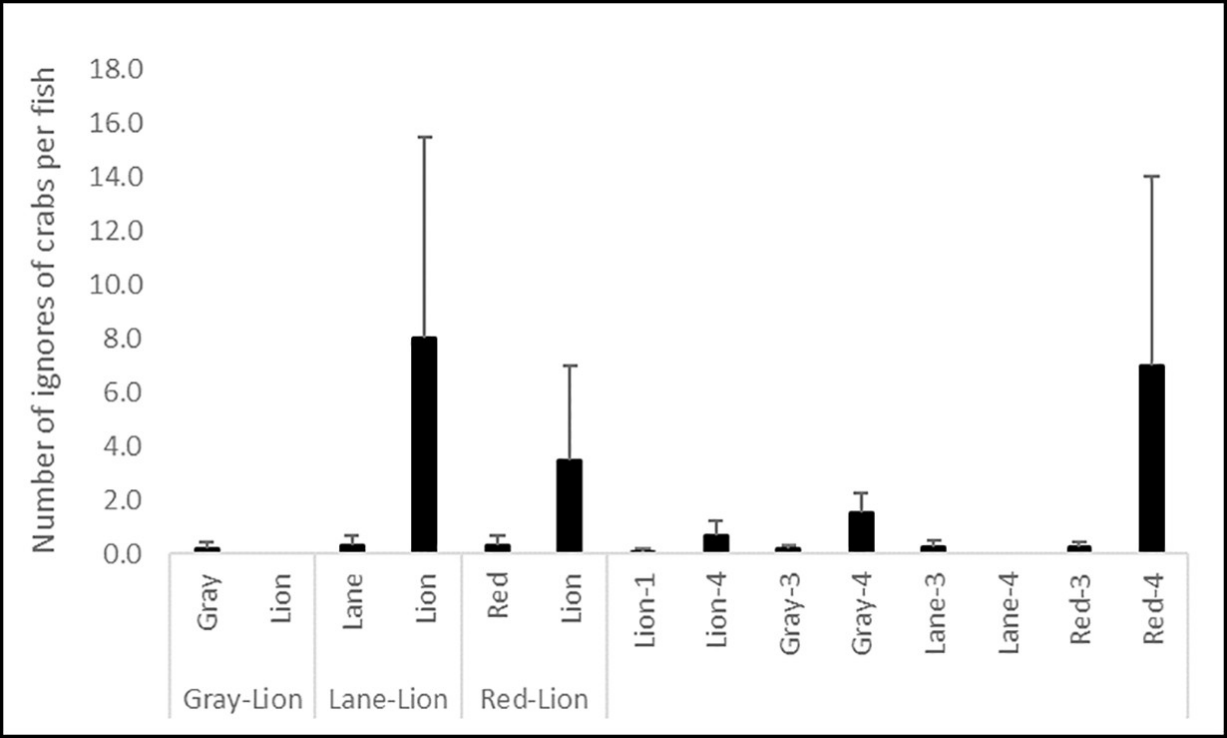
Number of ignores of nearby blue crab prey per fish (± 1 SE) by lionfish and red, lane, and gray snappers during control and interaction experiments. Data in the left portion of the figure are from interaction trials between lionfish and a given snapper species, and data in the right portion of the figure are from lionfish and snapper monoculture control trials of three and four individuals of a given snapper species (gray, lane, or red snapper), and one or four individuals for lionfish (lion).

No significant difference in the percentage of time spent pursuing prey (K = 20.080, df = 13, p = 0.093; Fig 5) or number of non-consumptive approaches by fishes toward prey (K = 20.388, df = 13, p = 0.086; Fig 6) were observed. However, both behaviors were more frequent for lionfish in all trials, and for red snapper and gray snapper in control trials. Few retreats from prey were observed, and while more frequent for red snapper and lionfish, no significant difference in ignoring passing prey by fishes (K = 11.044, df = 13, p = 0.607; Fig 7) existed among treatments.

**Fig 8:**
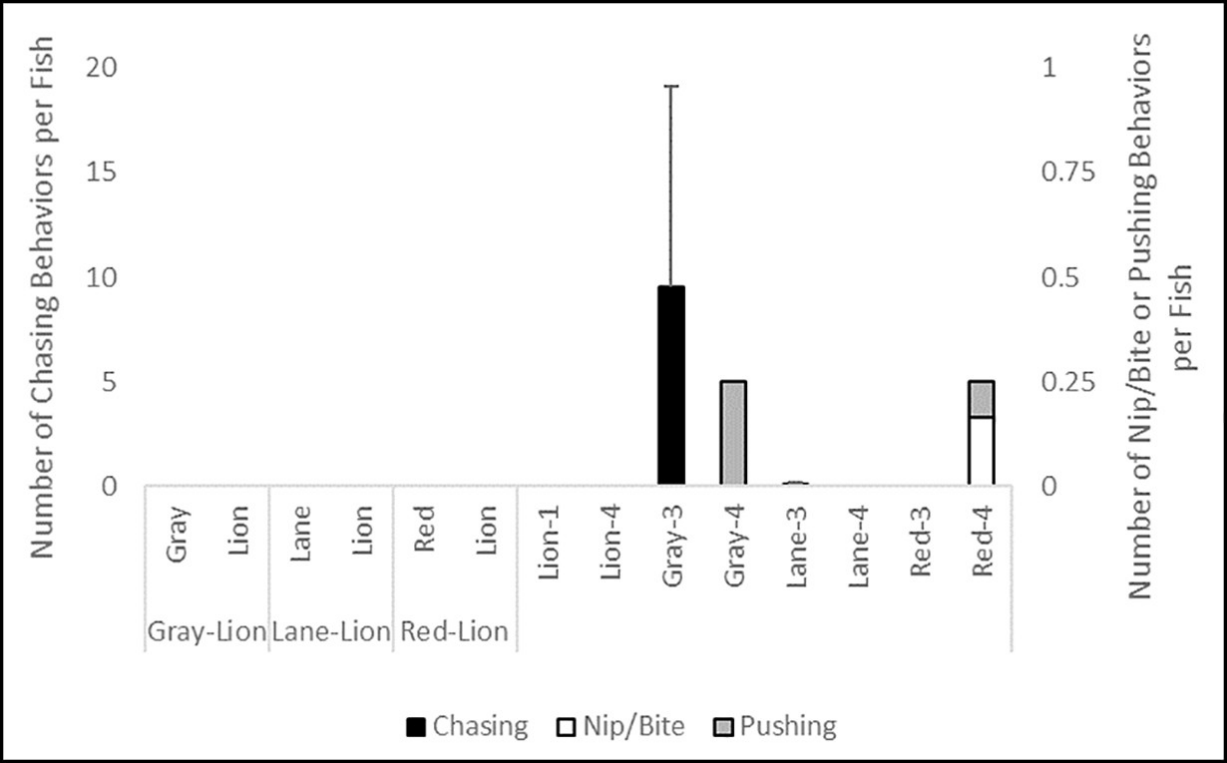
Number of inter- and intraspecific aggressive interactions observed per fish (± 1 SE) during control and interaction experiments. Data in the left portion of the figure are from interaction trials between lionfish and a given snapper species, and data in the right portion of the figure are from lionfish and snapper monoculture control trials of three and four individuals of a given snapper species (gray, lane, or red snapper), and one or four individuals for lionfish (lion).

No interspecific aggressions between lionfish and snappers were observed; however, intraspecific aggression among snappers occurred during control trials (Fig 8). While there was no significant difference in total aggressions per fish among treatments or species (K = 10.798, df = 13, p = 0.628), intraspecific chasing was the most frequently observed aggressive behavior between gray snappers, while nipping, biting, or pushing occurred very infrequently for all three snapper species.

No significant positive linear relationship between temperature (range: 14.4–30.5°C) and any fish behavior was observed, while the strongest relationship was found for all three environmental factors, average length, and swimming around the tank behavior (Table 5; R^2^ = 0.41). Relationships were polynomial, parabolic, or inverse parabolic. Similar weak relationships were observed between salinity (14.3–28.5) and dissolved oxygen (5.0–16.7 mg/L) on prey consumption and fish behaviors, with the only significant relationships found between average length and percent time huddling (R^2^ = 0.22; S1 Fig) and salinity and percent time interacting (R^2^ = 0.20; S1 Fig). Highest interaction times were observed for lionfish and gray snapper or lane snapper with salinity, while larger sized gray snapper in 4-individual controls or red snapper when paired with lionfish were observed most frequently huddling.

**Table 5:** Multiple regression relationships among environmental parameters, average fish total length (mm), and observed fish behaviors in snapper-lionfish control and interaction trials.

		p-value			
Fish Behavior	R^2^	Temperature	Salinity	Oxygen	Average Length
Swimming Around Tank	0.41	0.381	0.142	0.097	0.782
Swimming at Block/Edge	0.28	0.739	0.824	0.938	0.708
Number of Times Fish Ignored Prey	0.27	0.173	0.327	0.547	0.785
**Huddling**	0.22	0.200	0.702	0.551	**0.015**
**Percent Time Swimming Together**	0.20	0.287	**0.012**	0.112	0.075
Predatory Attempts	0.19	0.586	0.822	0.258	0.342
Consumption of Crabs	0.16	0.841	0.869	0.422	0.562
Approaches on Other Fishes	0.15	0.967	0.193	0.857	0.139
Total Aggressions	0.15	0.096	0.642	0.107	0.226
Percent Time Pursuing Prey	0.13	0.994	0.118	0.320	0.402
Prey Approaches	0.12	0.840	0.313	0.467	0.457
Swimming at Center	0.11	0.818	0.314	0.881	0.260
Lionfish Flaring Pectorals	0.10	0.368	0.916	0.274	0.454
Retreats from Other Fishes	0.10	0.421	0.647	0.473	0.451
Retreats from Prey	0.05	0.769	0.833	0.747	0.288

Values in bold indicate significant relationships (p<0.05) among factors and behaviors.

Additionally, no significant relationship was observed between time of day (mid-morning, afternoon, late afternoon) and behavioral variables (K = 0.522 to 6.306, df = 1 to 3, p = 0.062 to 0.648).

## Discussion

Examining interspecific competition between lionfish and three snapper species (as well as intraspecific competition within these three snapper species) has revealed that native-invasive competitive interactions in these fishes are complex and species-specific. We observed partial competitive advantages for lionfish in interactions with all three snapper species. These include lionfish being significantly more active than red and lane snapper, and consuming and attacking more prey than lane and gray snapper (S2 Table). Lionfish also approached snapper species more often than they retreated from them, but did not approach snappers significantly more often than snappers approached lionfish. In the presence of lionfish, red snapper were significantly less active than in controls, while no differences were observed in lane or gray snapper activities between control or lionfish-interaction trials. Although not statistically significant, red snapper also retreated more often than they approached lionfish, and were the only snapper species for which this was observed. These results suggest a differential competitive vulnerability and resistance by red, lane, and gray snapper to lionfish, as additionally inferred by nGOM field observations [49].

No interspecific aggressive behavior by lionfish was observed, suggesting that more efficient resource exploitation, intimidating appearance, and lack of aversion to other fish species may be more important than direct behavioral interference [66]. Significantly decreased swimming times by red and lane snappers in the presence of lionfish and significant approaches of lionfish toward these species appeared to confirm this suggestion. These findings complement additional studies demonstrating species-dependent effects of lionfish on fish swimming and foraging activities [67–68], and contribute additional information regarding lionfish behavioral effects on mesopredators. In this study, lionfish seemed to exploit resources more effectively than red and lane snappers, and restrict their swimming ranges compared to snappers. By conducting a controlled mesocosm study, we were able to obtain direct observations of the interactions between lionfish and native Gulf of Mexico fishes, complementing observations inferred and suggested from field studies [23, 49, 69]. Although variable, overall higher use of habitat (i.e., swimming around the tank) and prey consumption was observed for lionfish when paired with snapper species, while any interference competitive intensities were species-specific and limited to occasional aggressions between gray snapper individuals. However, gray snapper appeared to be less affected by lionfish, and our results suggested that they may be occasionally gregarious with lionfish in their interactions. These observations complement field studies relating nGOM snapper density to lionfish abundance [49] for which negative relationships were observed for red and lane snapper, but not gray snapper. Lionfish also flared their fins at red snapper significantly more often than other snapper species. A recent study has also shown that lionfish species flare fins at each other to initiate group hunting [70], but this behavior was not directly observed in other trials, which suggests that red snapper posed a stronger perceived threat to lionfish than lane or gray snapper. Based upon differential activity, swimming times, and prey consumption rates between paired and control trials, it appears that nGOM red, gray, and lane snapper each have a degree of partial competitive resistance to lionfish, while red and lane snapper appear to have higher competitive vulnerabilities to their invasion.

Given that the size of lionfish used in our experiments were similar to those observed in natural and artificial habitats [23], we are confident that our findings approximate natural ecological interactions within the nGOM. While larger lionfish were used in interactions with gray snapper than in lionfish controls, this size differential did not appear to strongly influence our results, as gray snapper behavior with lionfish did not significantly differ from that of other snappers and lionfish size was not strongly related to behavior. Additionally, gray snapper size influenced interactions with lionfish and their predatory attempts, with smaller individuals attacking prey more often and being observed under lionfish pectoral fins. These behaviors suggest no strong effect of lionfish size on gray snapper behavior, within the size ranges we used in this study, as also suggested by initial field observations [49]. Moreover, no significant differences among snapper sizes were found between interaction and control trials, or for snapper predatory attempts, suggesting overall minimal size effects among species, except for greater huddling activity among larger-sized individuals.

Additionally, while lionfish activity is generally greatest during crepuscular periods [31], time of day did not affect lionfish activity significantly during our experiments, while minimal environmental influence was observed with only a correlative association of interaction time with salinity. While salinity range differed with lionfish-lane snapper interaction trials, most effects appeared to be reflective of species behaviors than the influence of salinity. As other lane snapper behaviors were unaffected by salinity and a similar snapper interaction study found no influence of salinity on lane snapper behavior [42], we are confident that these differential interactions were not heavily influenced by salinity range, especially given no effect on lane snapper or lionfish swimming activities.

Our study suggests that lionfish activity can be affected by both hetero- and conspecific individuals, as documented in field studies [36–40], while intraspecific mechanisms, including density-dependence, acting upon lionfish have also been observed [71]. Additionally, our findings partially complement other studies, which have found little native resistance to lionfish [18, 29]. The partial competitive advantage of lionfish over red and gray snapper also aligns with observations that lionfish impacts on fish communities may be partial or mixed, depending on species [33–40]. In simulations, negative impacts of lionfish on small and intermediate sized carnivorous and omnivorous fishes and to crustaceans have been observed [72], while neutral and positive results on reef fish communities have also been observed [29, 33–34], potentially as a result of lionfish predation diminishing competitive interactions among prey fishes [29]. Similarly uncertain effects of lionfish on native species have been observed in Belize [73], and no evidence of negative effects by lionfish has been observed on medium-sized or larger-sized piscivores on Bahamian reefs [52] or throughout Belize [34]. Although reef fish species richness has declined at small scales due to lionfish consumption [27, 29, 74], no large changes in species or evenness have occurred as a result of their presence.

While our findings suggest some potential for lionfish to compete with nGOM snapper species, their effects are likely to occur at small scales. Our results indicate that lionfish consumption rates do not significantly differ from those of red snapper, which, like lionfish, also feed on a wide range of fauna [75]. Additional studies have demonstrated the importance of community composition, scale, and habitat when examining the impacts of lionfish. For example, no strong impacts of lionfish upon native fish communities or community stability in Los Roques, Venezuela have been detected, where lionfish were most often observed in areas of highest species richness and density, suggesting that fish community composition may be a major factor influencing the overall ecological effects of invasive lionfish [41]. Although the relationship between large grouper and lionfish abundance remains uncertain [33–35], high topographic complexity may explain their observed coexistence in some areas. Additionally, a smaller effect by lionfish has been observed in larger expanses of habitat [74], which suggests that lionfish impacts upon native fishes may be diluted over more continuous nGOM habitats.

In the nGOM, the highest densities of lionfish have been observed at artificial reefs [23] where their ecological effects are likely to be concentrated. Additionally, displacement of red snapper into higher portions of the water column by lionfish has been observed [69]. High topographic complexity and species richness in artificial systems may contribute to their attractiveness to lionfish, as observed for other nGOM species [46]. Although lionfish were initially observed in deeper, more structurally complex nGOM habitats [21], they also have moved into shallower, less complex areas [24, 76]. Progression of lionfish into seagrass and mangrove habitats has been observed [77], and while larger lionfish have been documented to migrate greater distances [25, 32], there is only circumstantial evidence that ontogenetic migrations occur between habitat types [78].

Although our study suggests only partial competitive advantage to lionfish, their effects on red snapper swimming activities (including huddling of larger individuals in the presence of lionfish) and partial effects on red snapper predation could allow for increased habitat partitioning, while their higher consumption rates could also be advantageous against lane and gray snapper. Increased lionfish predatory attempts and consumption rates in the presence of these two species when compared to 1-individual controls additionally suggests lessened perception of lane and gray snapper competitive potential than of red snapper by lionfish. Since the effects of overfishing continue to reduce red snapper size on artificial and natural reefs, lionfish may exacerbate the effects of fishing on snapper species [79]. This is because juvenile and early adult nGOM snappers appear partially vulnerable to lionfish presence, which can affect snapper predatory success and ability to occupy habitat. These outcomes can thereby reduce fitness and population viability over time, which can be compounded by fishing-related effects on size and reproductive output [79]. Additionally, as lionfish continue to move inshore onto lower-relief habitats that are home to smaller juvenile reef fishes, their negative effects could become more widespread. However, these effects are likely to be weaker in deep, more continuous habitats, which support high red snapper densities and serve as key recruitment areas [79–80]. Continued field and laboratory investigations regarding the effects of differential temperature, salinity, and substrate on lionfish-reef fish interactions are warranted and can build on our initial findings, especially as lionfish densities may differ in particular habitats beyond the 3:1 lionfish/snapper ratio investigated in our study.

Ongoing stock rebuilding and conservation efforts to enhance snapper populations are critical in combating the threats of biological invasions, regional warming, and overfishing. As lionfish culling efforts continue [49, 81–82], our findings suggest that concentrating these efforts on artificial reef and inshore habitats will likely be most beneficial, and have the strongest impact in diluting competitive impacts on native snapper species. Quantification of lionfish interactions is useful in elucidating the effects these invasive species may have, which will benefit ecosystem-based fisheries management and integrated ecosystem approaches in the Gulf of Mexico [83–85]. Ultimately, examining differential vulnerabilities to biological invasions can allow the formulation of strategies to mitigate effects on historically overfished commercially important species, such as red snapper.

## Supporting information

S1 Table Definitions of recorded fish behaviors in experimental mesocosms during snapper and lionfish control and interaction trials. (PDF)

S2 Table Summary of all behaviors and significant or non-significant differences among interaction and control trials for snappers and lionfish. (PDF)

S1 Fig Regression relationships between salinity and percent time interacting for all fishes and control and interaction trial types (top panel) and average total length (mm) and percent huddling time (bottom panel). (TIF)

S1 File Marshak et al. 2018, PLoS One Supporting Data. (XLSX)
