# Supplementary material for: Ecological interactions between Gulf of Mexico snappers (Teleostei: Lutjanidae) and invasive red lionfish (Pterois volitans)
Source: PLoS One. 2018 Nov 1;13(11):e0206749. doi: 10.1371/journal.pone.0206749 (PMC6211729; doi:10.1371/journal.pone.0206749)
Supplement: S1 Table — (PDF) [file pone.0206749.s001.pdf]

Term (Dependent Variable)	Definition
Percent time spent interacting (% trial time)	Percent time of the entire three-hour trial in which fishes of two or more species werein close proximity to each other (<20 cm), approached each other, or displayed aggressions to each other.
Number of lionfish pectoral fin flares	Number of observed pectoral fin flares at prey, a snapper, or another lionfish in a given interaction or control trial.
Percent time swimming tank (% trial time swimming)	Percent time of the three-hour trial in which at least one fish of a given species swam beyond the boundaries of concrete block habitat and covered ≥25% of the experimental mesocosm area in a given swim.
Percent time swimming block (% trial time swimming)	Percent time of the three-hour trial in which at least one fish of a given species swam or shifted at or around the perimeter of concrete block habitat, not exceeding 25% of the experimental mesocosm area in a given swim.
Percent time swimming at center (% trial time swimming)	Percent time of the three-hour trial in which at least one fish of a given species swam or shifted at or around the perimeter of the center of the mesocosm tank, not exceeding 25% of the mesocosm area in a given swim.
Percent time huddling (% trial time huddling)	Percent time of the three-hour trial in which at least two fish of a given species aggregated together, but did not swim, at the edge, center, or at concrete block habitat of the experimental mesocosm.
Number of approaches (or retreats)	Number of times a fish of a given species directly approached (or retreated from) a fish of another species.
Number of crabs consumed per fish	Number of blue crab prey observed partially or fully consumed by each fish of a given species.
Number of predatory attempts per fish	Number of times each fish attacked blue crab prey
Percent time pursuing prey (% trial time)	Percent time of the entire three-hour trial in which fish of a given species were observed following, approaching, stalking, or attempting to consume blue crab prey prior to successful consumption.

Number of prey approaches per fish	Number of times each fish followed, stalked, or actively moved within proximity of blue crab prey.
Number of retreats from prey per fish	Number of times each fish retreated from approaching blue crab prey.
Number of ignores of prey per fish	Number of times each fish did not respond to prey within close proximity, or to prey that passed directly in front of fish.
Number of aggressions per fish (chases, bites, pushes)	Number of times each fish chased another fish, nipped at or bit another fish, or pushed its body against another fish to cause the fish to react by moving in or from its location. Both intra-and interspecific aggressions were examined.
