# Supplementary material for: Ecological interactions between Gulf of Mexico snappers (Teleostei: Lutjanidae) and invasive red lionfish (Pterois volitans)
Source: PLoS One. 2018 Nov 1;13(11):e0206749. doi: 10.1371/journal.pone.0206749 (PMC6211729; doi:10.1371/journal.pone.0206749)
Supplement: S2 Table — (PDF) [file pone.0206749.s002.pdf]

Behavior	Significance	Gray Snapper	Lane Snapper	Red Snapper	Lionfish
Number of Lionfish pectoral fin flares	**Yes, at red snapper** (p=0.019)				Lionfish **flared at red snapper significantly more often** than at gray or lane snapper, **causing red snapper to pause swimming**.
Percent time swimming tank	**Yes** (p=0.002)	**No significant difference between gray snapper and lionfish** swimming times in interaction trials;	**Lane snapper swam significantly less than lionfish** in interaction trials;	**Red snapper swam significantly less than lionfish** in interaction trials;	**Lionfish swam significantly more than lane snapper** in interaction trials;
		**No significant difference among** gray snapper swimming times in gray-lionfish **interaction and** gray snapper **control trials**.	**No significant difference** among lane snapper swimming times in lane-lionfish **interaction and** lane snapper **control trials**.	**When paired with lionfish, red snapper swam significantly less than in** their 1-individual and 4-individual **control trials**.	**When paired with gray snapper, lionfish swam significantly less than in** their 1-individual **control trials**;<br><br>**No significant difference among** lionfish swimming times in lane-lionfish or red-lionfish **interaction and** lionfish **control trials**.

Behavior	Significance	Gray Snapper	Lane Snapper	Red Snapper	Lionfish
Percent time swimming block	**Yes** (p=0.005)	**Although minimal, gray and lane snapper swam significantly more at blocks than lionfish** in interaction trials;<br><br>**No significant difference among** gray or lane snapper swimming times at blocks in gray-lionfish or lane-lionfish **interaction** and gray or lane snapper **control trials**.		**Although minimal, red snapper swam significantly more at blocks than lionfish** in interaction trials;<br><br>**When paired with lionfish, red snapper swam significantly more at blocks than in** their 1-individual **control trials**.	**Lionfish swam significantly less** at blocks than gray, lane, and red snapper **in interaction trials**;<br><br>**No significant difference among** lionfish swimming times at blocks in **interaction and control trials**.
Percent time huddling	**Yes** (p=0.009)	**Although minimal, significantly higher huddling activity** for gray snapper **in gray snapper 4-individual control trials**.		**Although minimal, significantly highest huddling activity** for red snapper **in lionfish interaction trials**.	

Behavior	Significance	Gray Snapper	Lane Snapper	Red Snapper	Lionfish
Number of approaches (or retreats)	**Yes for lionfish** (p=0.034)	**No significant difference between gray, lane, and red snapper approaches and retreats from lionfish in interaction trials.**			**Lionfish approached all three snapper species more often than they retreated from them in interaction trials.**
Number of crabs consumed per fish<br><br>Number of predatory attempts per fish	**Yes** (p=0.007)<br><br>**Yes** (p=0.016)	**Gray snapper and lane snapper consumed and attempted to prey on significantly less crabs** than lionfish **in interaction trials**;<br><br>**No significant difference among** gray or snapper prey consumption or predatory attempts in gray-lionfish **interaction and** gray snapper **control trials**.		**No significant difference between red snapper and lionfish prey consumption or predatory attempts in interaction trials**;<br><br>**When paired with lionfish, red snapper consumed and attempted to prey upon significantly less crabs than in** their 3-individual **control trials**.	**Lionfish consumed and attempted to prey upon significantly more crabs** than gray and lane snapper **in interaction trials;**<br><br>**No significant difference between lionfish and red snapper prey consumption or predatory attempts in interaction trials;**

Behavior	Significance	Gray Snapper	Lane Snapper	Red Snapper	Lionfish
Number of crabs consumed per fish	**Yes** (p=0.007)				**When paired with lane or gray snapper, lionfish attempted to prey upon and consumed significantly more crabs than in** their 1-individual **control trials;**<br><br>**No significant difference among lionfish prey consumption or predatory attempts in** red-lionfish **interaction and** lionfish **control trials.**
Number of predatory attempts per fish	**Yes** (p=0.016)				
Percent time spent interacting	No (p=0.629)				
Percent time swimming at center	No (p=0.116)				
Percent time pursuing prey	No (p=0.093)				
Number of prey approaches (or retreats) per fish	No (p=0.086)				

Behavior	Significance	Gray Snapper	Lane Snapper	Red Snapper	Lionfish
Number of times prey was ignored per fish	No (p=0.607)				
Number of aggressions per fish	No (p=0.628)				
