# Supplementary figures and images for: Ecological interactions between Gulf of Mexico snappers (Teleostei: Lutjanidae) and invasive red lionfish (Pterois volitans)
Source: PLoS One. 2018 Nov 1;13(11):e0206749. doi: 10.1371/journal.pone.0206749 (PMC6211729; doi:10.1371/journal.pone.0206749)

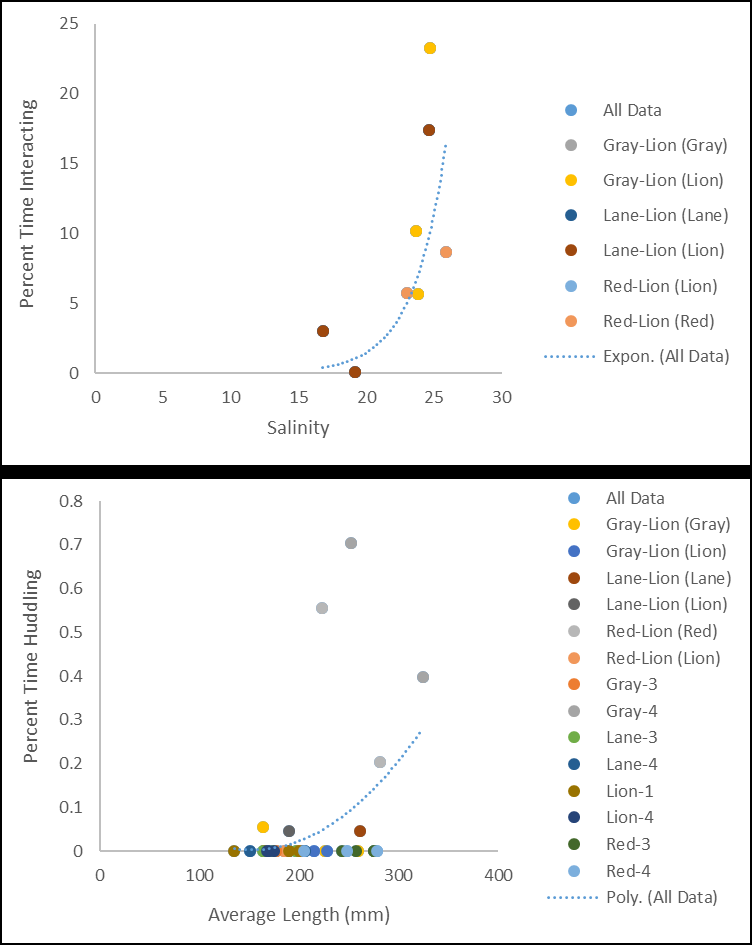

Supplement: S1 Fig — (TIF) [file pone.0206749.s003.tif]
